# LDL in patients with subclinical hypothyroidism shows increased lipid peroxidation

**DOI:** 10.1186/s12944-015-0092-4

**Published:** 2015-08-25

**Authors:** Kexi Zha, Changting Zuo, Aihong Wang, Bingchang Zhang, Yan Zhang, Bei Wang, Yunjia Wang, Jiajun Zhao, Ling Gao, Chao Xu

**Affiliations:** Department of Endocrinology, Shandong Provincial Hospital affiliated to Shandong University, Jinan, Shandong 250021 China; Department of gynaecology and obstetrics, Shandong Provincial Hospital affiliated to Shandong University, Jinan, Shandong 250021 China; Department of Cardiology, Shandong Provincial Hospital affiliated to Shandong University, Jinan, Shandong 250021 China; Clinical Laboratory, Shandong Provincial Hospital affiliated to Shandong University, Jinan, Shandong 250021 China; Scientific Center, Shandong Provincial Hospital affiliated to Shandong University, Jinan, Shandong 250021 China; Institute of Endocrinology and metabolism, Shandong Academy of Clinical Medicine, 324, Jing 5 Rd, Jinan, Shandong China; Department of endocrinology, Shanghai Second People’s Hospital, Shanghai, 250021 China; Department of endocrinology, Shuguang Hospital Baoshan Branch, Shanghai, 250021 China; Department of Health Care, Qianfoshan Hospital Affiliated to Shandong University, Jinan, 250014 China

**Keywords:** Subclinical hypothyroidism, Oxidative stress, Low density lipoproteins

## Abstract

**Background:**

Population-based studies have demonstrated that subclinical hypothyroidism (SCH) is an independent risk factor for atherosclerosis (OR = 1.9). However, this connection cannot be entirely explained by dyslipidemia accompanied by SCH. Lipid peroxidation also plays an important role in the development of atherosclerosis. In this study, we aimed to evaluate oxidative stress in SCH patients, as measured according to concentrations of hydroxy-octadecadienoic acids (HODEs) and hydroxy-eicosatetraenoic acids (HETEs) in both plasma and low density lipoproteins (LDL).

**Subjects and methods:**

The concentrations of HODEs and HETEs in both LDL and plasma were examined in euthyroid (*n* = 10), mild SCH (4.5 ≤ TSH <10 mU/L, *n* = 10), and significant SCH (TSH ≥ 10 mU/L, *n* = 10) subjects, using a liquid chromatograph-electrospray ionization- mass spectrometer. Then, we explored the relationship among LDL oxidation, TSH levels, and carotid intima-media thickness (IMT), a biomarker of subclinical atherosclerosis.

**Results:**

Serum LDL-C levels and mean-IMT in the significant SCH group were higher than in the euthyroid group (*p* < 0.05). The HODE and HETE concentrations clearly increased in the significant SCH patients compared with the euthyroid subjects, but there was no difference between the mild SCH and euthyroid groups. Among all subjects, linear and significant positive correlations were identified between TSH and mean-IMT after adjustment for confounding factors (r = 0.480, *p* = 0.018). Both 9-HODE (r = 0.376, *p* = 0.041) and 13-HODE (r = 0.447, *p* = 0.013) in LDL were linearly and positively correlated with TSH. The concentrations of HODEs (both 9-HODE and 13-HODE) in LDL were much higher in the thickened IMT group than in the normal IMT group (*p* = 0.017 and 0.015, respectively). HODEs in LDL were also positively associated with mean-IMT.

**Conclusions:**

Our findings showed that lipid peroxidation was higher in the significant SCH patients than in the euthyroid subjects, which suggested that qualitative as well as quantitative changes in serum lipids resulting from SCH may add to atherosclerosis risk.

**Electronic supplementary material:**

The online version of this article (doi:10.1186/s12944-015-0092-4) contains supplementary material, which is available to authorized users.

## Background

Subclinical hypothyroidism (SCH), which is defined as elevated serum thyroid-stimulating hormone (TSH) and normal serum free thyroxine (FT4), is becoming a global health problem because of its increasing prevalence and potential deleterious effects. SCH occurs in 4–20 % of the adult population, even more than the population with diabetes mellitus and is increasing globally. Moreover, population-based studies demonstrate that both the risk and mortality of coronary heart disease are increased in SCH patients [1–3]. The reason may be that SCH is an independent risk factor for atherosclerosis (OR = 1.9), which was demonstrated in the Rotterdam Study [4]. Although SCH is often accompanied by dyslipidemia, the increased concentration of serum lipids does not necessarily result in atherosclerosis [5]. Indeed, the quantitative alteration of lipid profiles in SCH patients was still controversial [6, 7]. Increasing experimental and epidemiological evidence shows that lipid peroxidation plays an important role in atherosclerosis development. However, few studies have evaluated oxidation in SCH patients.

The oxidation of LDL converts it into an atherogenic form that contributes to the development of the atherosclerotic lesion. One of the major pathways of LDL oxidation is the lipoxygenase pathway by seeding molecules that include hydroperoxyoctadecadienoic acid (HPODE) and hydroperoxyeicosatetraenoic acid (HPETE), which are subsequently reduced to their hydroxyl derivatives hydroxy-octadecadienoic acids (HODEs) and hydroxy-eicosatetraenoic acids (HETEs) [8, 9]. Notably, these HODEs and HETEs enhance atherogenicity [10, 11] and are considered reliable oxidative stress biomarkers. Currently, the evidence supporting increased oxidative stress in LDL among SCH patients is scarce and indirect, mostly based on increased plasma autoantibody levels against oxidatively modified LDL [12]. To our knowledge, there are no data available on the alteration of HODEs and HETEs in either LDL or plasma in SCH patients.

In the present study, we attempted to explore the status of lipid peroxidation as measured by concentrations of HODE and HETEs. We further explored the relationships among lipid oxidation, TSH levels, and carotid intima-media thickness (IMT), a biomarker of subclinical atherosclerosis [13]. In addition, we compared the differences in HODE and HETEs levels in plasma and LDL, respectively, attempting to identify a better surrogate marker for IMT. Our findings may also help fully elucidate the mechanism that underlies the association between high atherosclerosis risk and SCH from a new perspective.

## Results

### Characteristics of the study groups

The clinical and laboratory data are depicted in Table 1. According to their serum TSH concentrations, the subjects were divided into 3 groups: euthyroid, mild SCH, and significant SCH. There was no significant difference among the 3 groups with regard to serum FT3 or FT4 levels. Serum LDL-C levels and mean-IMT in the significant SCH group were higher than they were in the euthyroid group (*p* < 0.05), whereas no differences were found between the mild SCH and euthyroid groups (*p* > 0.05). There were no significant differences between groups for other factors as well, including age, gender distribution, BMI, systolic and diastolic pressure, fasting plasma glucose, TC, HDL-C, and TG levels (*p* > 0.05).

**Table 1:** Clinical and laboratory data of the studied groups

Variable	Euthyroid	Mild SCH	Significant SCH	*p* ^a^	*p* ^b^	*p* ^c^
n	10	10	10	NA	NA	NA
Gender (male/female)	4/6	3/7	2/8		0.879	
Age (years)	52.0 ± 5.7	53.2 ± 5.4	57.3 ± 5.5	1.000	0.125	0.329
BMI (kg/m^2^)	24.0 ± 1.6	24.4 ± 1.8	24.4 ± 1.5	1.000	1.000	1.000
SBP (mmHg)	130.0 ± 17.8	129.6 ± 12.4	126.4 ± 21.5	1.000	1.000	1.000
DBP (mmHg)	77.1 ± 8.5	80.4 ± 6.8	75.3 ± 5.6	0.915	1.000	0.353
Mean-IMT (mm)	0.75 ± 0.09	0.82 ± 0.14	0.99 ± 0.32	1.000	0.045	0.193
FBG (mmol/l)	5.41 ± 0.63	5.72 ± 0.65	5.34 ± 0.31	0.477	0.946	0.202
FT3 (pmol/L)	4.79 ± 0.39	5.23 ± 0.77	4.89 ± 0.41	0.210	0.825	0.376
FT4 (pmol/L)	14.33 ± 1.42	14.55 ± 1.59	13.79 ± 1.47	1.000	1.000	0.652
TSH (mIU/L)	3.32 ± 0.57	5.92 ± 1.47	12.98 ± 3.23	0.013	0.000	0.000
TC (mmol/l)	5.15 ± 0.43	5.75 ± 1.00	5.81 ± 1.13	0.117	0.183	0.990
HDL-C (mmol/l)	1.45 ± 0.15	1.50 ± 0.36	1.61 ± 0.42	0.896	0.429	0.762
LDL-C (mmol/l)	2.78 ± 0.35	3.16 ± 0.61	3.43 ± 0.59	0.372	0.032	0.779
TG (mmol/l)	0.92 ± 0.07	1.16 ± 0.27	1.15 ± 0.31	0.117	0.142	1.000

Continuous and categorical variables data are expressed as the mean ± standard deviations and real number of subjects, respectively. The statistical *p* value was generated by one-way ANOVA with Bonferroni correction as the post-hoc test. The χ-square test was employed to compare gender distribution. *P* ^*a*^represents the mild SCH *vs* euthyroid group, *P* ^*b*^represents the significant SCH *vs* euthyroid group, and *P* ^*c*^represents the significant SCH *vs* mild SCH group. Abbreviation: mild SH, mild subclinical hypothyroidism group; significant SCH, significant subclinical hypothyroidism group; *BMI* body mass index, *mean-IMT* mean carotid intima-media thickness, *FBG* fasting blood glucose, *FT3* free T3, *FT4* free T4, *TSH* thyroid stimulating hormone, *TC* total cholesterol, *HDL-C* high-density lipoprotein, *LDL-C* low-density lipoprotein, *TG* triglyceride, *NA* not applicable

As shown in Additional file 1: Table S1, the TC content in LDL increased gradually but not significantly among the euthyroid, mild SCH, and significant SCH groups (*p* > 0.05). The TG content in LDL was also not significantly different.

### A positive relationship between TSH and mean-IMT levels

**Fig. 1:**
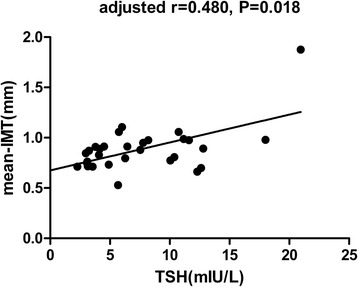
Correlation analysis between TSH and mean-IMT in the study population. Partial correlation analysis after adjustment for age, gender, BMI, TC, HDL-C, LDL-C and TG: *r* = 0.480, *p* =0.018

Carotid IMT is strongly associated with atherosclerosis. An IMT greater than 0.9 mm is almost certainly indicative of atherosclerosis and increased risk of cardiovascular disease [14]. Notably, among all subjects, linear and significant positive correlations were identified between TSH and mean-IMT (*r* = 0.401, *p* = 0.028). Interestingly, the correlations remained significant even after we adjusted for age, gender, BMI, TC, HDL-C,LDL-C, and TG (*r* = 0.480, *p* = 0.018) (Fig. 1). This finding suggests an association between SCH and the risk for atherosclerosis.

### Lipid peroxidation increased in SCH patients

Lipid peroxidation plays an important role in developing atherosclerosis. Notably, HETEs and HODEs are the stable end products of lipid peroxidation. Subsequently, we determined the HODEs and HETEs in plasma and LDL, respectively among the euthyroid, mild SCH, and significant SCH groups.

**Fig. 2:**
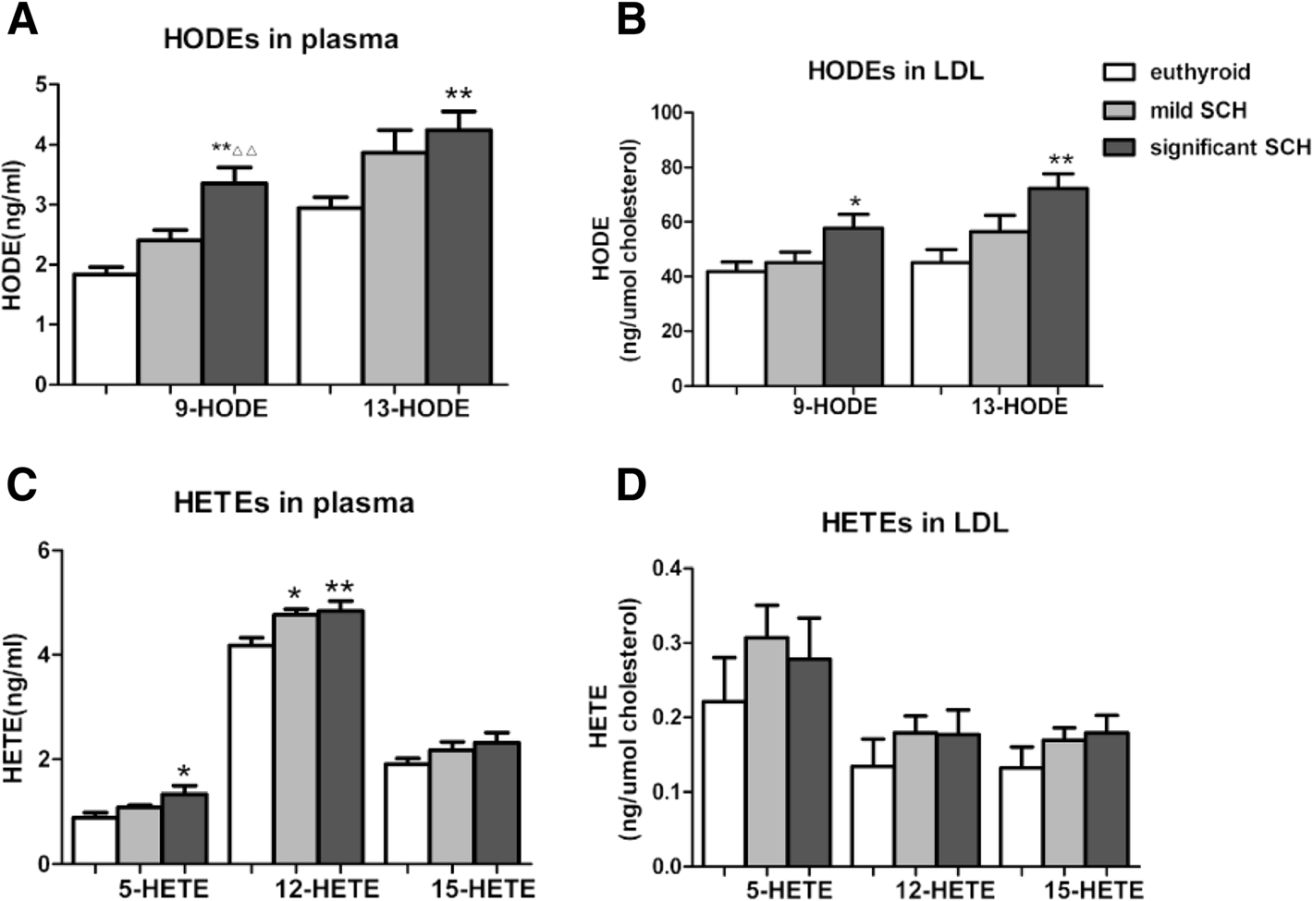
HODEs and HETEs in plasma and LDL respectively among the euthyroid, mild SCH, and significant SCH groups. The concentrations of 9- and 13- HODE in plasma (**a**) and in LDL (**b**) among the euthyroid, mild SCH, and significant SCH groups; the concentrations of 5-, 12- and 15-HETE in plasma (**c**) and in LDL (**d**) among the euthyroid, mild SCH, and significant SCH groups. Data are means ± SEM (*n* = 10 participants per group). Compare with euthyroid subjects: *p < 0.05 **p < 0.01 mild SCH compare with significant SCH :△*p* < 0.05 △△*p* < 0.01. Abbreviation: mild SCH, mild subclinical hypothyroidism group; significant SCH, significant subclinical hypothyroidism group; HODE, hydroxy-octadecadienoic acid; HETE, hydroxy-eicosatetraenoic acid

As shown in Fig. 2, the concentrations of HODEs (both 9-HODE and 13-HODE) in plasma clearly increased in the significant SCH patients compared with the euthyroid subjects, and there was no difference between the mild SCH and euthyroid groups. Similar findings were also obtained with respect to the HODE concentrations in LDL. However, the results for the HETE concentrations in plasma were not consistent with those for LDL. Compared with the euthyroid subjects, plasma 5-HETE and 12-HETE levels in the significant SCH patients were higher. Notably, no significant differences were detected with regard to the HETE concentrations in LDL among the 3 groups.

### HODEs in LDL were positively associated with TSH

**Fig. 3:**
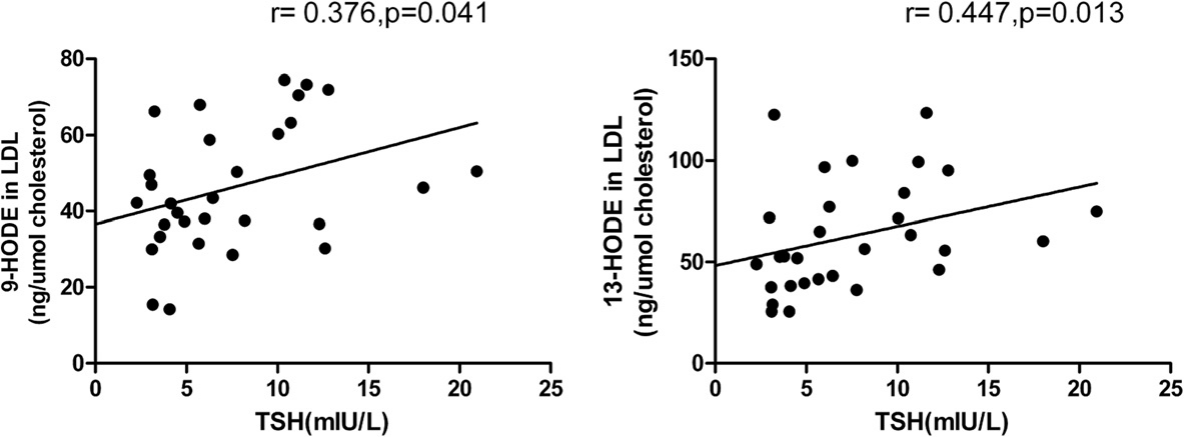
Correlation analysis between HODEs in LDL and TSH in the study population. Spearman’s correlation analysis indicated TSH levels were linearly and positively correlated with 9-HODE in LDL (*r* = 0.376, *p* = 0.041) and 13-HODE in LDL (*r* = 0.447, *p* = 0.013) respectively. *Abbreviation: HODE*, hydroxy-octadecadienoic acid

Then, we evaluated the relationship between HODEs in LDL and TSH. As shown in Fig. 3, the Spearman’s correlation analysis revealed that both 9-HODE (*r* = 0.376, *p* = 0.041) and 13-HODE (*r* = 0.447, *p* = 0.013) in LDL were linearly and positively correlated with TSH, which indicated that lipid peroxidation in LDL was significantly aggravated with the elevation of TSH.

### HODEs in LDL might be more sensitively indicative of atherosclerosis

Then, we detected the concentrations of HETEs and HODEs in both plasma and LDL to evaluate their role in the indication of atherosclerosis.

As mentioned above, we divided all subjects into two groups according to their IMT: normal (max-IMT ≤ 0.9 mm) (*n* = 16) and thickened (max-IMT > 0.9 mm) (*n* = 14). Notably, the HODE concentrations (both 9-HODE and 13-HODE) in LDL were much higher in the thickened IMT group than they were in the normal group (Additional file 2: Figure S1, *p* = 0.017 and 0.015, respectively). No difference was found in plasma HODE levels between the normal and thickened IMT groups. Moreover, there were no significant differences in the HETE concentrations (5-, 12-, and 15-HETE) in either plasma or LDL between the normal and thickened IMT groups (data not shown).

In addition, we also evaluated the relationship between HODEs in LDL and mean-IMT using Spearman’s correlation analysis (Additional file 3: Figure S2). Interestingly, a positive and significant correlation was obtained between mean-IMT and the concentration of 9-HODE in LDL (*r* = 0.479, *p* = 0.007); as well as the concentration of 13-HODE in LDL (*r* = 0.447, *p* = 0.013).

These data suggested that (1) compared with HETEs, HODEs were a more reliable biomarker for atherosclerosis and (2) compared with HODEs in plasma, HODEs in LDL might be more sensitively indicative of atherosclerosis.

## Discussion

Our study confirmed that SCH, especially significant SCH (TSH ≥ 10 mIU/L), was associated with a high risk of atherosclerosis. Indeed, previous studies have suggested the connection between SCH and atherosclerosis. However, this connection cannot be entirely explained by dyslipidemia accompanied by SCH. Our findings seem to present a more plausible explanation for this connection from a new perspective.

Consistent with our results, population-based studies have demonstrated that SCH is an independent risk factor for atherosclerosis (OR = 1.9) and myocardial infarction (OR = 3.1) [4]. However, the changes in the lipid profiles of SCH patients were still controversial. Although some studies have demonstrated that total cholesterol and LDL-C levels are elevated in patients with SCH [6], others have not shown any effect of SCH on these lipid measurements. In the National Health and Nutrition Examination Survey III (*n* = 8586), SCH was not associated with changes in serum cholesterol or triglyceride levels after adjustment for confounding factors [7]. In a cross-sectional study of 7000 thyroid clinic outpatients, total cholesterol and LDL-C were clearly elevated in overt hypothyroid patients, but there were no significant differences in serum total cholesterol, LDL-C, HDL-C, or triglyceride levels between SCH patients and the euthyroid control group [15]. Therefore, the association between SCH and atherosclerosis cannot be entirely explained by dyslipidemia accompanied by SCH. Our findings showed that LDL-C levels and lipid peroxidation markedly increased mainly in significant SCH patients compared with euthyroid subjects, which suggested that qualitative as well as quantitative changes in serum lipids resulting from SCH may add to atherosclerosis risk.

To the best of our knowledge, data on oxidative stress in SCH patients are still limited and controversial. In one study, SCH patients were found to be associated with increased oxidative stress manifested by reduced arylesterase activity and elevated catalase (free-radical scavenging enzyme) activity. Arylesterase, one of the enzymatic activities of paraoxonase-1, is known to play a protective role against the peroxidation of LDL and other lipoproteins. However, no significant correlations were observed after TC levels were controlled for. Accordingly, oxidative stress in SCH seemed to be induced by hypercholesterolemia secondary to thyroid dysfunction but not hypothyroidism *per se* [16]. Two other studies showed that malondialdehyde, a lipid peroxidation marker, was increased in SCH patients, which suggested increased oxidative stress in SCH states [17, 18]. Additionally, superoxide dismutase (free-radical scavenging enzyme) levels were found to be higher in SCH patients and shown to decrease after L-T4 treatment for 3 months [19]. Nevertheless, little is known about LDL susceptibility to oxidation in SCH patients and its relation to TSH levels. Here, we further determined the lipid peroxidation markers, mainly HETEs and HODEs, in both LDL and plasma in SCH patients and euthyroid controls.

Our data revealed that 9-HODE and 13-HODE, derived from linoleic acid, significantly increased in significant SCH patients in both plasma and LDL compared with those of the control subjects. Meanwhile, the concentrations of 5-HETE and 12-HETE were significantly increased in SCH patients compared with control subjects only in plasma but not in LDL. These results were in agreement with those from previous studies. Patients with essential hypertension have been reported to show increased plasma 13-HODE levels, presumably reflecting increased oxidative stress [20]. Recently, Colas et al. [21] demonstrated that oxidation products of linoleic acid (HODEs) could be better markers of lipid peroxidation than those of arachidonic acid, such as total HETEs and F2-isoprostanes in LDL in obese patients with metabolic syndrome. Linoleic acid occurs at a ratio of roughly 7:1 compared with arachidonic acid in the LDL particle [22] and therefore may be the predominant target for peroxidation. Meanwhile, HETEs are more prone to further oxidation than HODEs. Because of the high levels of linoleic acid and high stability of 9- and 13-HODE, these two hydroxy acids are enriched in naturally occurring lipid peroxidation processes to a greater extent than any other lipid peroxidation products and are nearly ideal markers for lipid peroxidation [23, 24]. Notably, our results also showed that HODEs in LDL were positively associated with TSH and might be more reliably and sensitively indicative of atherosclerosis, which might substantiate evidence for an association among lipid peroxidation, SCH, and atherosclerosis.

The pathogenesis of atherosclerosis in SCH patients can be partially explained by the increased 9- and 13-HODE in LDL. Nagy et al. identified 9-HODE and 13-HODE, two of the major oxidized lipid components of oxLDL, as endogenous activators and ligands of PPARγ, which regulates the expression of CD36 in macrophages [25]. This suggested that 9- and 13- HODE may play a direct role in the regulation of macrophage gene expression during atherogenesis. Additionally, 9- and 13-HODE reduce monocyte CCR2 expression through pathways involving PPARγ, which may help retain monocytes at sites of inflammation such as atherosclerotic lesions. This may accelerate atherogenesis [26]. In contrast, native linoleic acid without oxidative modification had no effect. Increased HODE levels thus contribute to atherosclerosis progression and the risk of clinical events such as myocardial infarction or stroke. Except for HODEs, Wigren et al. reported that 5- and 12-HETE could also activate PPARγ but were less potent [11]. Therefore, the increase in multiple HODEs and HETEs in LDL or plasma in patients with SCH might present possible mechanisms for atherosclerosis in these patients. Additional studies are required to explore whether TSH can regulate the production of HODEs or HETEs.

Similar to a recent study [21], we found no significant change in LDL levels of 5-,12- and 15-HETE in SCH patients compared with those of the euthyroid subjects, although the exact mechanism is not clear. Proudfoot et al. [27] reported that HDL is the major lipoprotein carrier of plasma F2-isoprostanes (oxidation products of arachidonic acids, just as HETEs) and that F2-isoprostanes were significantly higher in HDL than in LDL or VLDL after isolation via ultracentrifugation or FPLC. As for HETEs, it is also necessary to determine the distribution in different lipoproteins, which will be helpful for elucidating the disparity of increased HETE levels in plasma rather than in LDL in SCH patients.

The limitations of this study are the small sample size and lipid peroxidation has not been performed after treatment to subclinical hypothyroidsm. It is possible that the enhanced lipid peroxidation noted may revert to normal following correction of hypothyroidism with thyroxine treatment. But, this needs to be established.

Taken together, our findings showed that lipid peroxidation was markedly higher in significant SCH patients than in euthyroid subjects, which suggested that qualitative as well as quantitative changes in serum lipids resulting from SCH may add to atherosclerosis risk. More prospective studies are required to assess the effect of maintaining TSH at normal levels on lipid abnormalities, peroxidation, and atherosclerosis.

## Materials and methods

### Subjects

The diagnosis of SCH was based on finding high TSH levels associated with normal FT4 levels. Depending on the size of the increase in the serum TSH, subclinical hypothyroidism can be classified as mild (serum TSH concentrations of 4.5–10 mU/L) or significant (TSH ≥10 mU/L) [28]. Based on the exclusion criteria [17] (diabetes mellitus, hypertension, obesity (body mass index, BMI ≥ 28 kg/m^2^), ischemic heart disease, history of or current smoking, active infection, malignancy, pituitary and rheumatologic diseases, and usage of drugs that affect the oxidant state or lipid parameters), we first included 160 newly diagnosed SCH patients from Ningyang County, Shandong Province, from January to February 2013. Patients’ thyroid function was measured twice for 6 months interval to rule out laboratory error or transient increases. Only 129 patients met the criteria of having two consistent results and a final diagnosis of SCH. Among these, we selected 20 SCH patients aged between 18 and 65 years using the random number table method: 10 mild SCH patients and 10 age- and sex- matched significant SCH patients. The control group consisted of 10 age- and sex-matched healthy euthyroid subjects from the same district. Histories, physical examinations, electrocardiography, and routine chemical analysis revealed that the control subjects showed no evidence of any disease. The study was performed according to the Declaration of Helsinki. All subjects signed an informed consent, validated and approved by the ethics committee of Shandong Provincial Hospital affiliated to Shandong University. Ethics committee of Shandong Provincial Hospital affiliated to Shandong University specifically approved this study (approval number: NO. 2013–105) (Additional file 4: Figure S3).

### Laboratory analysis

All of the measurements were performed in the clinical laboratory of Shandong Provincial Hospital, affiliated with Shandong University. Blood samples were collected from all patients between 8:00 A.M. and 10:00 A.M. after a minimum of a 10-h fast. For LDL isolation and monohydroxylated fatty acid detection, plasma was separated via centrifugation at 1500 g for 10 min with blood collected on EDTA. Then, 10 μM BHT was added to plasma and immediately frozen at −80 °C under nitrogen for further characterizations.

Chemiluminescent procedures (Cobas E610; Roche, Basel, Switzerland) were employed to determine thyroid function, including TSH, free triiodothyronine (FT3) and free tetraiodothyronine (FT4). The laboratory reference ranges were 0.27–4.2 mIU/L for TSH, 3.1–6.8 pmol/L for FT3, and 12–22 pmol/L for FT4. The levels of plasma glucose, total cholesterol (TC), triglyceride (TG), low-density lipoprotein cholesterol (LDL-C), and high-density lipoprotein cholesterol (HDL-C) were determined using an Auto Biochemical Analyzer (MODULAR-000GS; Roche, Basel, Switzerland). Hypercholesterolemia was defined as a TC value over 6.21 mmol/L, which is in accordance with the National Cholesterol Education Program Adult Treatment Panel III criteria (NCEP/ATPIII).

### Carotid artery ultrasound

All patients underwent a carotid IMT examination using a color ultrasonic diagnostic apparatus equipped with a 9 MHz linear-array transducer (Toshiba Aplio 500 Ultrasound Scanner) as described previously [29]. The carotid IMT was defined as the viewable distance between the blood-intimae and the media-adventitia interfaces on the artery wall. Mean IMT (mean-IMT: the mean of the three IMT measurements on each side) and the maximum IMT (max-IMT: the highest IMT value among the six segments studied) were assessed. According to current sonographic criteria, max-IMT ≤ 0.9 mm was normal, and max-IMT > 0.9 mm was considered indicative of thickened intima [29]. The study protocol was based on current sonographic guidelines [30]. Each scan was made by the same investigator, who was blind to the patients’ clinical data.

### LDL isolation using FPLC

LDL was isolated from plasma using gel filtration chromatography at 4 °C as previously described [31]. Briefly, a AKTA purifier system fast–protein liquid chromatography (FPLC) equipped with a Superose 6 10/300GL column (GE Healthcare, Pittsburgh, PA, USA) was used with a phosphate-buffered saline solution containing 1 mM EDTA and 0.02 % NaN_3_ as a running buffer. After filtered plasma was loaded, chromatography was conducted with a flow rate of 0.5 mL/min under a pressure of 218 psi. Fractions of 0.5 mL were collected, and the concentrations of TC and TG in the eluted fractions were measured (Applygen Technologies Inc, Beijing). The identified FPLC peaks are shown in Additional file 5: Figure S4. The LDL fractions were concentrated on a Millipore filter 30,000 MW (10 min at 4000 g). Then, the protein concentrations were determined using commercial kits (Biocolor BioScience & Technology Company, Shanghai). Enzymatic determination of TC and TG used commercial kits (Applygen Technologies Inc, Beijing).

### Quantification of total monohydroxylated fatty acids using LC-ESI-MS

In order to quantify total monohydroxylated fatty acid levels in plasma and LDL respectively, we prepared samples as described previously [32]. Briefly, following the lipid extraction of plasma and LDL using a Folch solution (chloroform-methanol, 2:1, v/v) containing 0.005 % (w/v) BHT, dried extracts were subjected to methanol hydrolysis with 1 M KOH for 30 min at 37 °C. After the PH of the mixture was adjusting to 3.0 by adding 5 N HCl, we added 5 ng 15(S)-HETE-d8 and 10 ng 13(S)-HODE-d4 as internal standards after the mixture had cooled to room temperature. After liquid-liquid extraction twice using ethyl acetate, the two organic phases were combined and the solvent was evaporated. Then, the residue was reconstituted into 100 μl of 80 % (v/v) LC mobile phase A(2 mM NH4Ac, PH5.6) and 20 % (v/v) B(CH3CN:MeOH,65:35,v/v). The solution was filtered using a Spin-X filter and was then ready for LC-ESI-MS analysis.

Mass spectrometry was performed on a Thermo Vantage triple-stage quadruple mass spectrometer (Thermo Fisher Scientific, San Jose, CA, USA) equipped with electrospray ionization (ESI).

Chromatographs were separated on a Thermo UltiMate3000 series (Thermo Fisher Scientific, San Jose, CA, USA). Chromatography was performed using a Unitary C 18 column (2.8 μm particle, 100 × 2.1 mm; Acchrom Technologies Co., Ltd). Mobile phase A consisted of 2 mM NH4Ac in water (PH 5.6), and mobile phase B consisted of 65 % acetonitrile in methanol (v/v). The auotosampler was set at 4 °C. The injection volume was 5 μl. The flow rate was controlled at 0.2 mL/min. The gradient program was as follows: 0–1 min, 20 % B; 1–20 min, linear gradient from 20 to 50 % B; 20–25 min, 50 % B; 25–30 min, linear gradient from 50–80 % B; 30–32 min, 80 % B; 32–35 min, linear gradient from 80–100 % B; 35–37 min 100 % B; 37–38 min, linear gradient from 100–20 % B; and 38–45 min 20 % B. The data acquisition and instrument control were accomplished using Xcalibur software version 1.4 (Thermo Fisher Scientific, San Jose, CA, USA). The MS was operated in negative ion mode using selective reaction monitoring (SRM) by monitoring the characteristic fragmentations. The transfer tube temperature was set at 450 °C. The flow rate of the sheath gas was 55 arb. The ion spray voltage was set at 4,500 V. The flow rate of the auxiliary gas was 25 arb. Collision energy, declustering potential and collision cell exit potential were optimized for each compound to obtain optimum sensitivity. The transitions monitored were mass-to-charge ratio (m/z): 295.0 → 171.0 for 9-HODE; 295.1 → 194.8 for 13-HODE; 299.0 → 197.9 for 13(S)-HODE-d4; 319.1 → 115.0 for 5-HETE; 319.0 → 179.0 for 12-HETE; 319.1 → 175.0 for 15-HETE; 327.1 → 226.1 for 15(S)-HETE-d8 (Additional file 6: Figure S5).

### Statistical analysis

Statistical analyses were performed with SPSS version 17.0 for windows. One-way ANOVA with Bonferroni correction as the post-hoc test was used to seek differences in variables among groups. Significance was defined as a value of *p* < 0.05. If unstated, all values are expressed as the mean ± SD for continuous variables. Correlation coefficients were determined using the Spearman correlation test.

## Additional files

Additional file 1: Table S1. Lipid classes of LDL from euthyroid subjects and mild and significant SCH patients. (DOC 46 kb) Additional file 2: Figure S1. Compare of 9- and 13- HODE in plasma and LDL in the normal and thickened intima groups respectively. A and B: Compared of 9- and 13-HODE levels in plasma in the normal (*n* = 16) and thickened intima (*n* = 14) groups; C and D: Compared of 9- and 13-HODE levels in LDL in the normal (*n* = 16) and thickened intima (*n* = 14) groups. Abbreviation: HODE, hydroxy-octadecadienoic acid; IMT, carotid intima-media thickness. (TIFF 420 kb) Additional file 3: Figure S2. Correlation analysis between HODEs in LDL and the mean-IMT levels in total study subjects. Spearman’s correlation analysis indicated a positive and significant correlation between mean-IMT and the concentration of 9-HODE in LDL (*r* = 0.479, *p* = 0.007) and 13-HODE in LDL (*r* = 0.447, *p* = 0.013) respectively. Abbreviation: HODE, hydroxy-octadecadienoic acid; mean-IMT, mean carotid intima-media thicknes. (TIFF 164 kb) Additional file 4: Figure S3. Screening procedure of total studied subjects. The final study group consisted of 10 cases with mild SCH, 10 cases with significant SCH and 10 age- and sex- matched euthyroid subjects. Abbreviation: mild SCH, mild subclinical hypothyroidism group; significant SCH, significant subclinical hypothyroidism group. (TIFF 2641 kb) Additional file 5: Figure S4. FPLC elution profiles for a representative plasma sample from a hypercholesterolemic complicated with hypertriglyceridemic individual. A: The elution volume, in milliliters, is represented on the χ-axis and the UV absorbance units (mAU), measured at 280 nm, are represented by the y-axis. B: The elution volume, in milliliters, is represented on the χ-axis and the concentrations of TC and TG are represented by the y-axis. The elution volume for VLDL ranges from 7 to 9 ml, from 9 to 13 ml for LDL, and from 13 ml to 17.5 ml for HDL. (TIFF 563 kb) Additional file 6: Figure S5. SRM of HODEs and HETEs from plasma or LDL. The transitions monitored were mass-to-charge ratio (m/z): 295.0 → 171.0 for 9-HODE; 295.1 → 194.8 for 13-HODE; 299.0 → 197.9 for 13(S)-HODE-d4; 319.1 → 115.0 for 5-HETE; 319.0 → 179.0 for 12-HETE; 319.1 → 175.0 for 15-HETE; 327.1 → 226.1 for 15(S)-HETE-d8. (TIFF 1255 kb)

## Abbreviations

SCH: Subclinical hypothyroidism
HODEs: Hydroxy-octadecadienoic acids
HETEs: Hydroxy-eicosatetraenoic acids
HPODE: Hydroperoxyoctadecadienoic acid
HPETE: Hydroperoxyeicosatetraenoic acid
IMT: Intima-media thickness
TSH: Thyroid-stimulating hormone
FT4: Free thyroxine
FT3: Free triiodothyronine
FBG: Fasting blood glucose
TC: Total cholesterol
TG: Triglyceride
LDL-C: Low-density lipoprotein cholesterol
HDL-C: High-density lipoprotein cholesterol
FPLC: Fast–protein liquid chromatography
LC-ESI-MS: Liquid chromatograph-electrospray ionization- mass spectrometer
