# Supplementary material for: LDL in patients with subclinical hypothyroidism shows increased lipid peroxidation
Source: Lipids Health Dis. 2015 Aug 25;14:95. doi: 10.1186/s12944-015-0092-4 (PMC4548906; doi:10.1186/s12944-015-0092-4)
Supplement: Additional file 1: Table S1. — Lipid classes of LDL from euthyroid subjects and mild and significant SCH patients. (DOC 46 kb) [file 12944_2015_92_MOESM1_ESM.doc]

**Additional table 1. Lipid classes of LDL from euthyroid subjects and mild and significant SCH patients.**

| Variable | Euthyroid | Mild SCH | Significant SCH | *p* ^a^ | *p* ^b^ | *p* ^c^ |
| --- | --- | --- | --- | --- | --- | --- |
| TC  (nmol/mg protein) | 384.87±103.64 | 425.42±88.34 | 487.72±192.96 | 0.733 | 0.179 | 0.316 |
| TG  (nmol/mg protein) | 204.21±31.85 | 214.04±56.36 | 193.84±86.38 | 0.914 | 0.905 | 0.475 |

Data are expressed as the mean±standard deviations. The statistical *p* value was generated by the one-way ANOVA with Bonferroni correction as the post-hoc test. *P ^a^* represents the mild SCH *vs* euthyroid group, *P ^b^* represents the significant SCH *vs* euthyroid group, and *P ^c^* represents the significant SCH *vs* mild SCH group. Abbreviation: mild SH, mild subclinical hypothyroidism group; significant SCH, significant subclinical hypothyroidism group.
