# Supplementary figures and images for: LDL in patients with subclinical hypothyroidism shows increased lipid peroxidation
Source: Lipids Health Dis. 2015 Aug 25;14:95. doi: 10.1186/s12944-015-0092-4 (PMC4548906; doi:10.1186/s12944-015-0092-4)

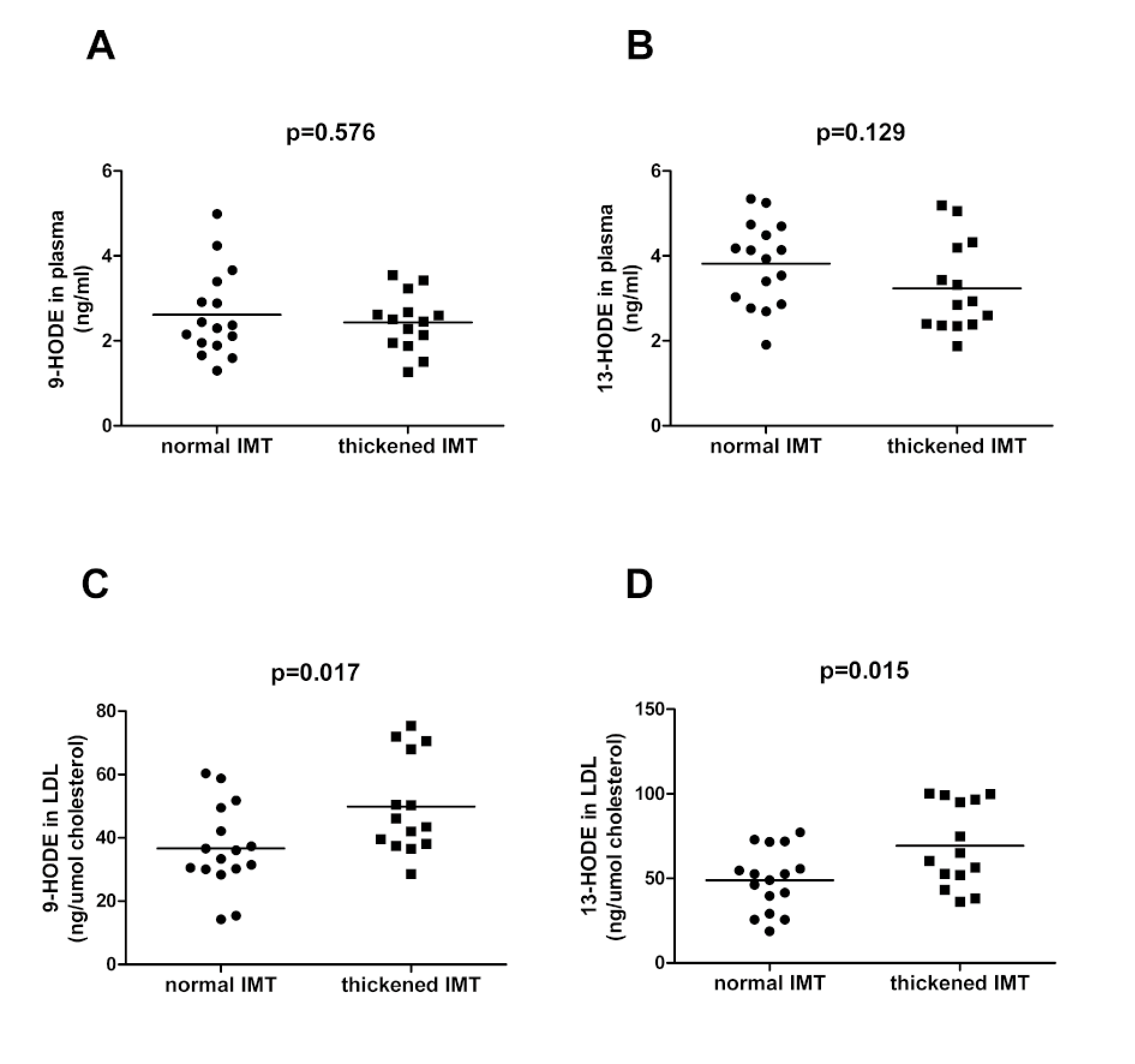

Supplement: Additional file 2: Figure S1. — Compare of 9- and 13- HODE in plasma and LDL in the normal and thickened intima groups respectively. A and B: Compared of 9- and 13-HODE levels in plasma in the normal (n = 16) and thickened intima (n = 14) groups; C and D: Compared of 9- and 13-HODE levels in LDL in the normal (n = 16) and thickened intima (n = 14) groups. Abbreviation: HODE, hydroxy-octadecadienoic acid; IMT, carotid intima-media thickness. (TIFF 420 kb) [file 12944_2015_92_MOESM2_ESM.tiff]

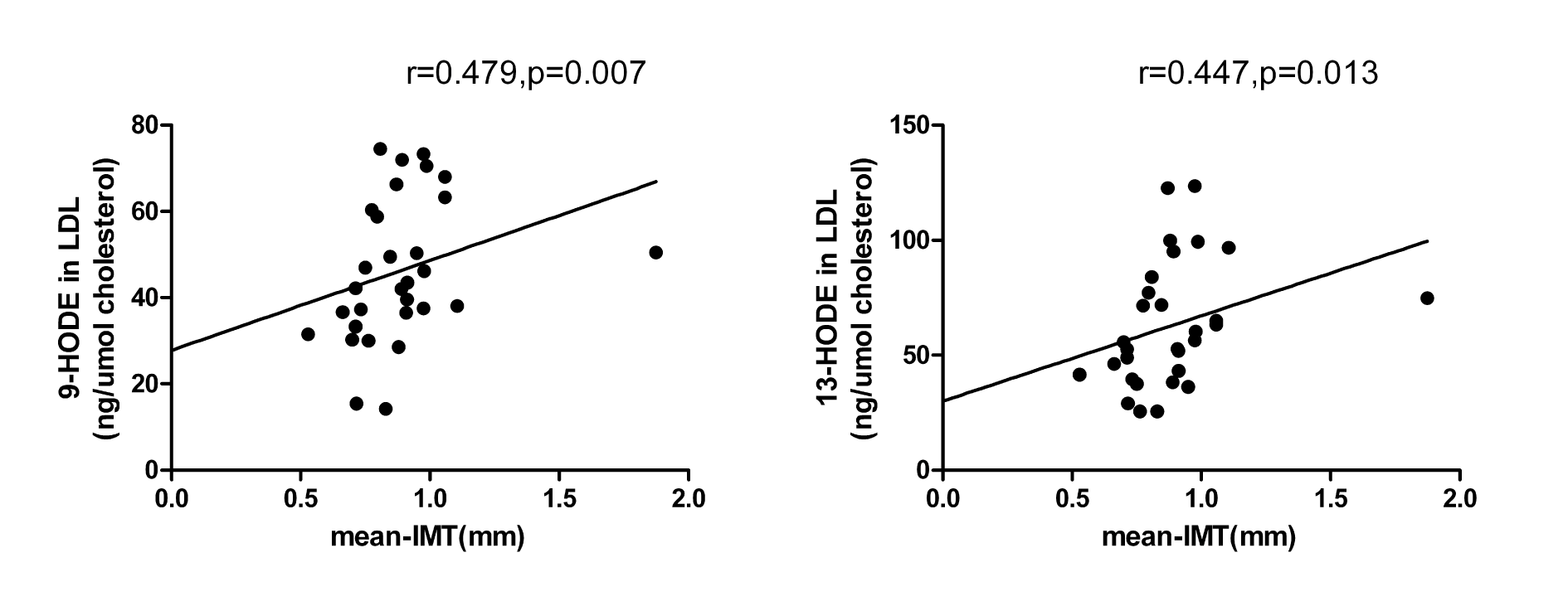

Supplement: Additional file 3: Figure S2. — Correlation analysis between HODEs in LDL and the mean-IMT levels in total study subjects. Spearman’s correlation analysis indicated a positive and significant correlation between mean-IMT and the concentration of 9-HODE in LDL (r = 0.479, p = 0.007) and 13-HODE in LDL (r = 0.447, p = 0.013) respectively. Abbreviation: HODE, hydroxy-octadecadienoic acid; mean-IMT, mean carotid intima-media thicknes. (TIFF 164 kb) [file 12944_2015_92_MOESM3_ESM.tiff]

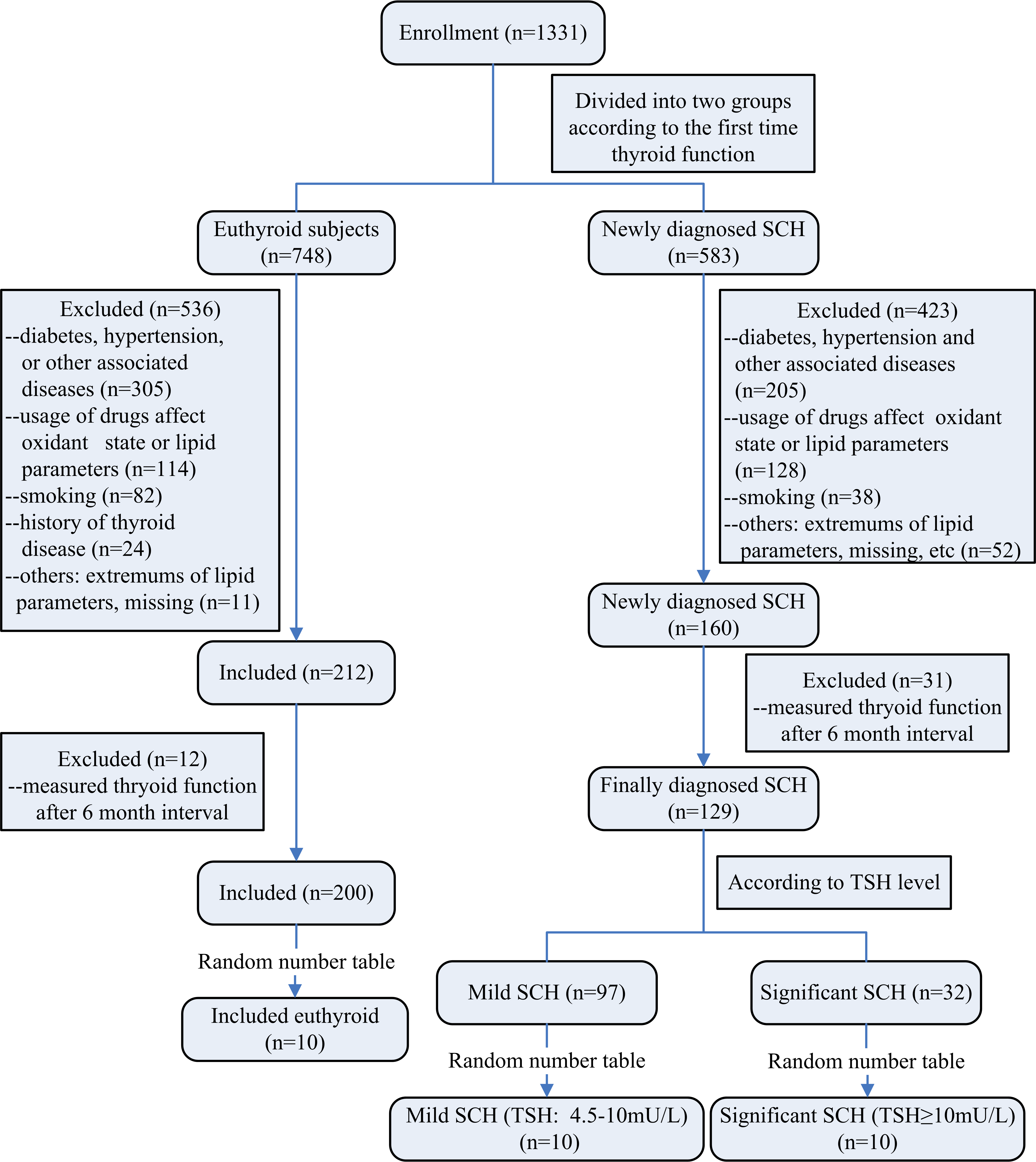

Supplement: Additional file 4: Figure S3. — Screening procedure of total studied subjects. The final study group consisted of 10 cases with mild SCH, 10 cases with significant SCH and 10 age- and sex- matched euthyroid subjects. Abbreviation: mild SCH, mild subclinical hypothyroidism group; significant SCH, significant subclinical hypothyroidism group. (TIFF 2641 kb) [file 12944_2015_92_MOESM4_ESM.tiff]

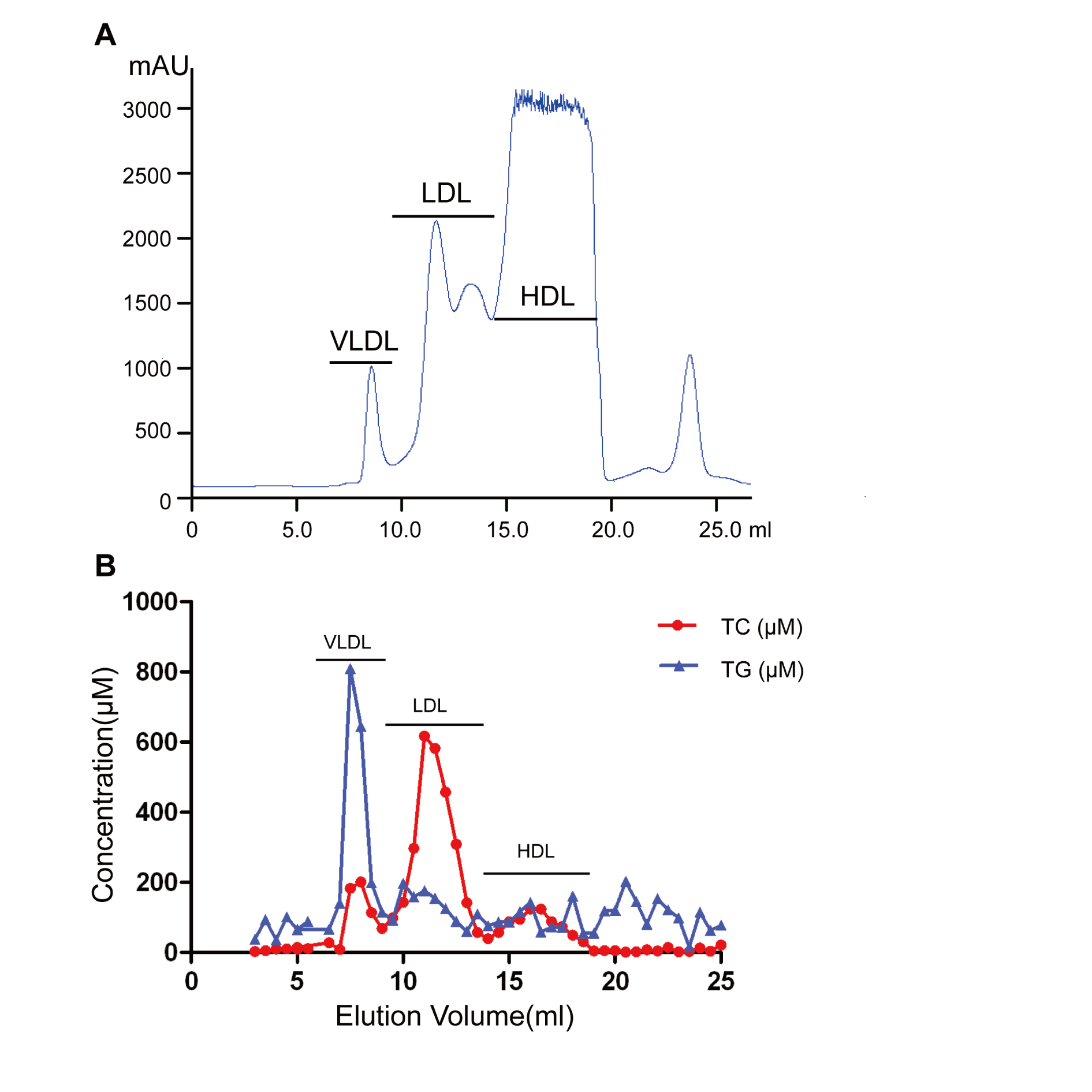

Supplement: Additional file 5: Figure S4. — FPLC elution profiles for a representative plasma sample from a hypercholesterolemic complicated with hypertriglyceridemic individual. A: The elution volume, in milliliters, is represented on the χ-axis and the UV absorbance units (mAU), measured at 280 nm, are represented by the y-axis. B: The elution volume, in milliliters, is represented on the χ-axis and the concentrations of TC and TG are represented by the y-axis. The elution volume for VLDL ranges from 7 to 9 ml, from 9 to 13 ml for LDL, and from 13 ml to 17.5 ml for HDL. (TIFF 563 kb) [file 12944_2015_92_MOESM5_ESM.tiff]

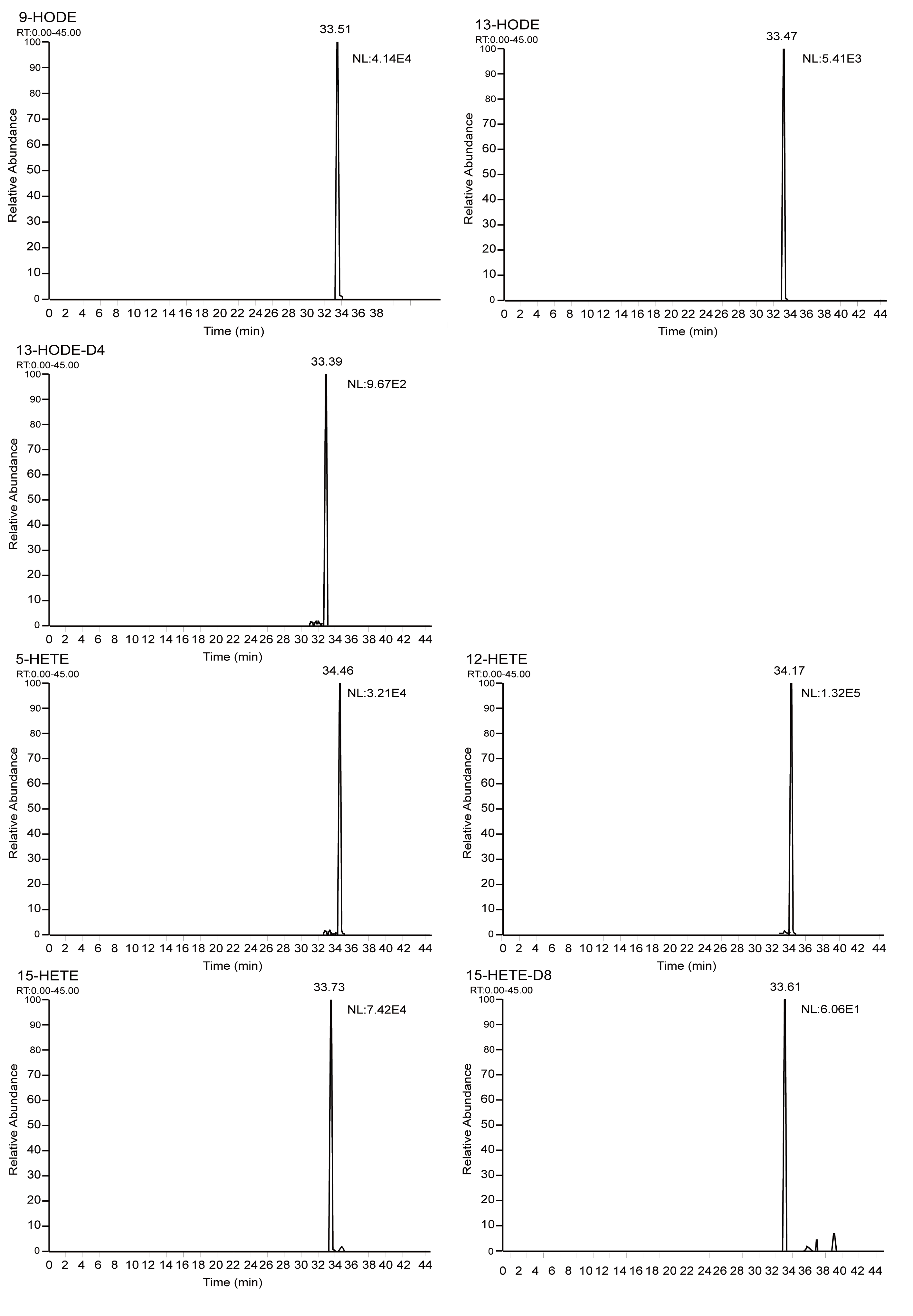

Supplement: Additional file 6: Figure S5. — SRM of HODEs and HETEs from plasma or LDL. The transitions monitored were mass-to-charge ratio (m/z): 295.0 → 171.0 for 9-HODE; 295.1 → 194.8 for 13-HODE; 299.0 → 197.9 for 13(S)-HODE-d4; 319.1 → 115.0 for 5-HETE; 319.0 → 179.0 for 12-HETE; 319.1 → 175.0 for 15-HETE; 327.1 → 226.1 for 15(S)-HETE-d8. (TIFF 1255 kb) [file 12944_2015_92_MOESM6_ESM.tiff]
